# Warwickshire's Record

**Published:** 1920-11-06

**Authors:** R. Hooper

**Affiliations:** Secretary


### Warwickshire's Record.

*To the Editor of* The Hospital.

Sir,—You may be interested to learn that the Hospital Saturday Committee's weekly collections among the employees of works, shops, collieries, etc., for the Coventry and Warwickshire Hospital during the past twelve months reached the unprecedented sum of £15,000. Last year the amounted collected was £8,350, but during the year an urgent appeal was made by their Committee for the weekly contributions to be increased. This met with a ready response, the majority agreeing to pay 2d. or 3d. per week or even a 1d. in the £. A special levy was also appealed for for one week of 1s. for adults and 6d. for girls and juniors. The result itself shows the success which these appeals met with. Many of the smaller firms, who hitherto have not been contributors, are now subscribing, and the shop assistants are also joining in. It is anticipated that before the end of another twelve months all employees in the city will be contributing. Even now the school teachers and members of office staffs are taking up the practice.—Yours faithfully,

November 1, 1920. R. Hooper, Secretary.

